# Timing of Heparin Administration Modulates Arterial Occlusive Thrombotic Response in Rats

**DOI:** 10.3390/jcdd7010010

**Published:** 2020-03-18

**Authors:** Amanda B. Matrai, Bryn Kastetter, Brian C. Cooley

**Affiliations:** 1 McAllister Heart Institute, University of North Carolina at Chapel Hill, Chapel Hill, NC 27599, USA; amandamatrai@hotmail.com (A.B.M.); bryn.kastetter@gmail.com (B.K.); 2 Department of Pathology and Laboratory Medicine, University of North Carolina, Chapel Hill, NC 27599, USA

**Keywords:** arterial thrombosis, anticoagulation, heparin, animal model

## Abstract

Background: The timing for initiation of effective antithrombotic therapy relative to the onset of arterial thrombosis may influence outcomes. This report investigates the hypothesis that early administration of heparin anticoagulation relative to the onset of thrombotic occlusion will effect a reduction in occlusion. Methods: A standard rat model of experimental thrombosis induction was used, injuring the carotid artery exposure with FeCl_3_-saturated filter paper, followed by flow monitoring for onset of occlusion and subsequent embolization events. Intravenous heparin administration (200 units/mL) was timed relative to the initiation of injury or onset of near occlusion, compared with controls (no heparin administration). Results: No occlusion was found for delivery of heparin 5 min prior to thrombus induction, whereas all vessels occluded without heparin. Unstable (embolic) thrombi were seen with heparin given at or shortly after initial occlusion. Only 9% (1/11) of the vessels had permanent occlusion when heparin was given at the time of thrombotic onset (*p* < 0.0001 vs. unheparinized), while 50% occluded when heparin was delayed by 5 min (*p* > 0.05). Conclusions: These findings provide evidence that antithrombotic therapy may need to be administered prior to the onset of anticipated loss of patency, with less effectiveness when given after occlusion has occurred.

## 1. Introduction

The onset of arterial thrombosis leading to vascular occlusion and cardiac ischemia is often difficult to assess. Furthermore, how quickly thrombogenesis progresses to occlusion once it is initiated is another unknown. These questions are relevant to plaque rupture leading to myocardial infarction and to interventions such as stenting or bypass grafting of the coronary artery. Antithrombotic therapies may be in place at the time of thrombus initiation; however, the specific timing of administration relative to when thrombogenesis is present may influence the development of an occlusive thrombus [1,2,3,4,5]. This is relevant in the above areas of practice where the decision to add an antithrombotic agent such as unfractionated heparin or bivalirudin may be made late, during or after reflow following an interventional procedure when thrombogenesis may well be underway [6,7]. We sought to address a simple aspect of these issues, using the timing of therapeutic heparin administration relative to the initiation of a vascular injury that leads to consistent thrombotic occlusion.

The ferric-chloride-based model of thrombosis induction was originally developed in rats [8]. Subsequently, it has been used extensively in mice, as a mouse model of arterial injury to induce thrombosis, to exploit the diversity of genetic models developed in mice [9,10]. However, with the advent of CRISPR (clustered regularly interspaced short palindromic repeats) and related gene-editing technologies, opening the way for genetic manipulations in many other species including rats, there may well be a return to using this thrombosis model more frequently in rats. We selected this model both because of its consistent induction of thrombotic occlusion and to further characterize its utility for studying the timing of therapeutic initiation relative to thrombotic occlusion. When anticoagulation is given, it may have a critical effect upon thrombotic occlusion, such that late administration may be ineffective, whereas more immediate administration may show a more favorable response. The major purpose of this study was to identify such a differential response in the timing of antithrombotic therapy, using standard unfractionated heparin.

The basic ferric chloride model of thrombosis involves application of ferric chloride to the surface of an artery, which generates a free-radical-based injury transferred to the lumenal surface, disrupting the endothelium and inducing a thrombotic response [11,12]. The model is straightforward and relatively easy to do from a technical perspective; however, there is limited data that can be acquired from it as practiced. The main, and often only, outcome measure recorded is the “time to occlusion” (TTO), the time point at which flow falls to zero or some near-zero consistent point (e.g., 25% of baseline flow). The assay is generally done for 30 min, but once occlusion occurs, the assay is often defined as completed. It has been noted by some investigators using the mouse version of the model [13,14] that the occlusive thrombus can subsequently embolize, resulting in reflow; this is a reflection of clot instability, a potentially important characteristic of the thrombus. This may have relevance to a microvascular repair site such as in coronary artery bypass grafting (CABG), from which a thrombus may undergo micro-embolic dissolution with apparently little detrimental downstream effect and result in subsequent maintained patency [15]. To evaluate the potential for this embolic phenomenon with the rat ferric chloride model, an experimental series was carried out using moderate-dose heparin infusion to demonstrate the utility of expanding the outcome measures in the rat version of the FeCl_3_ model.

## 2. Materials and Methods

Adult male Lewis rats (320–450 g) were obtained from Charles Rivers Laboratories (38 total). The animals were maintained under controlled environmental conditions (12 h light/dark cycle) with free access to food and water. All procedures were approved by the Institutional Animal Care and Use Committee of UNC Chapel Hill.

### 2.1. Establishment of Vessel Injury Model

The FeCl_3_ method used was essentially done as first described by Kurz and colleagues [8] with some modifications. Rats were anesthetized via intraperitoneal injection of 50 mg/Kg pentobarbital followed by the exposure and cannulation of the saphenous vein for intravenous drug administration. A midline ventral incision was made from the mandible to the suprasternal notch, and a segment of the right common carotid artery was exposed using blunt dissection. After a brief irrigation with 0.9% saline and wicking away the irrigant, a piece of filter paper (2 × 4 mm) was saturated with a 40% FeCl_3_ solution (Sigma Aldrich, St. Louis, MO, USA, dissolved in distilled H_2_O) and applied to the adventitial surface of the vessel for 3 min; this approach was known to induce 100% thrombotic occlusion in control rats. The filter paper was subsequently removed, and the vessel was extensively washed with saline. Blood flow was monitored with an ultrasonic flow probe (Transonic Systems, Ithaca, NY, USA) placed on the artery. The time to thrombotic occlusion (TTO) was defined as the time at which a fall to 25% of the initial baseline flow occurred, with a subsequent continued decline towards zero flow. Flow was continuously monitored for 30 min to identify subsequent reflow events. A reflow event, indicative of an embolism, was recorded as the time to reflow (TTR) if the flow returned above 10% of baseline with a continued increase in flow beyond 25% of baseline.

### 2.2. Experimental Design

To induce antithrombotic activity, heparin (Heparin Sodium Injection, Sagent Pharmaceuticals, Schaumburg, IL) was infused in a total amount of 200 IU/kg under differently timed intravenous bolus infusion strategies. The dose was selected based on similarity to high-dose clinical use and previous experience using heparin for preventing thrombosis in rats [16,17,18]. Pre-Heparin (*n* = 6): Heparin was injected 5 min before FeCl_3_ injury; TTO Heparin (*n* = 11): Heparin was injected when flow reached 25% of baseline; TTO + 5 min Heparin (*n* = 10): Heparin was injected 5 min after flow reached 25% of baseline; Saline Control (*n* = 11): Rats were infused with physiological saline when flow reached 25% of baseline. The timing of infusions relative to the onset of thrombus induction made the study not conducive to experimenter blinding.

### 2.3. Assessment

**Figure 1:**
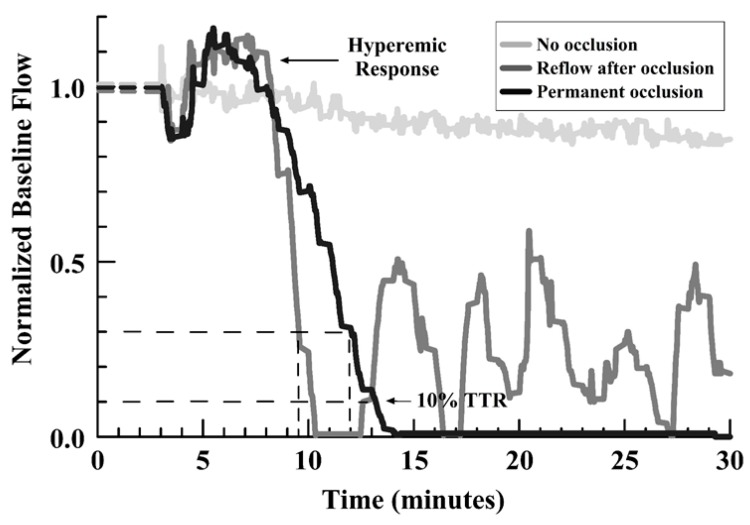
Line graph of representative flow outcomes after FeCl_3_ injury, showing no occlusion, permanent occlusion, and reflow events after initial occlusion (see legend in graph).

The main outcome measures were: 1) the initial TTO (at 25% of baseline flow), 2) the development or absence of any occlusion, and 3) the occurrence of embolism/reflow after occlusion (evidence of an unstable thrombus), and 4) the TTR for a first embolism relative to the TTO. Figure 1 shows representative curves for those encountered, identifying the types of outcome measures.

### 2.4. Statistical Analysis:

TTO and TTR data were analyzed with Kruskal–Wallis among-groups comparisons, with Mann–Whitney nonparametric tests for between-groups comparisons, using a *p* < 0.05 level to define significance. Incidence of occlusion and reflow were analyzed by Fisher Exact tests (2 × 3 table), using the same criterion for significance.

## 3. Results

**Figure 2:**
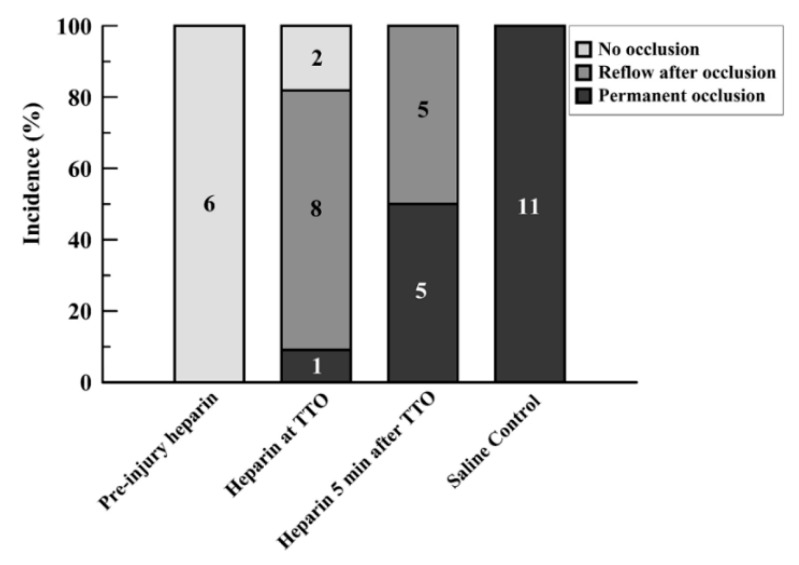
Bar graph showing incidence of events after FeCl_3_ injury for the four study groups. Events were separated into three categories: no occlusion, reflow after occlusion, and permanent occlusion. Values are expressed in percent proportion, with the numbers within bars showing the number of runs for each group with each particular event criterion.

Preinjury injection of heparin led to sustained flow without occlusion in all rats (6/6; Group 1; Supplemental Figure S1). Injection of heparin at the time of identified onset of occlusion (TTO) (Group 2) caused an immediate reversal in the drop in flow in two of the rats, with continued patency above 25% baseline flow. Another 8 of 11 in this group had reflow/embolism events after occlusion, which often had subsequent reocclusion and re-embolism (Figure 2). Only 1 of 11 had a permanent occlusion by the established criteria, although this vessel also showed slight reflow events of under 25% baseline flow after initial occlusion (Supplemental Figure S2). When the heparin injection was delayed for 5 min after the TTO (Group 3), 5 out of 10 rats (50%) maintained permanent occlusion; with reflow events in the other 5 rats (Supplemental Figure S3). The control series, with saline injected at the TTO, resulted in permanent occlusion in all vessels (11/11; Figure 2 and Supplemental Figure S4).

Giving heparin at the time of occlusion (Group 2) yielded statistically significant improved reflow versus controls (Group 4; *p* < 0.0001) but not for later heparin infusion (Group 3; *p* = 0.0513) despite an apparent difference (Figure 2). Heparin given before injury (Group 1) versus at later times (Groups 2 and 3) resulted in significantly better patency (*p* = 0.0045 and *p* < 0.0001, respectively; Fisher Exact tests). Controls (no heparin; Group 4) had greater occlusion rates than the other three groups, including late heparin ((*p* < 0.01 vs. Groups 1 and 2, and *p* = 0.0124 vs. Group 3; Fisher Exact tests).

**Figure 3:**
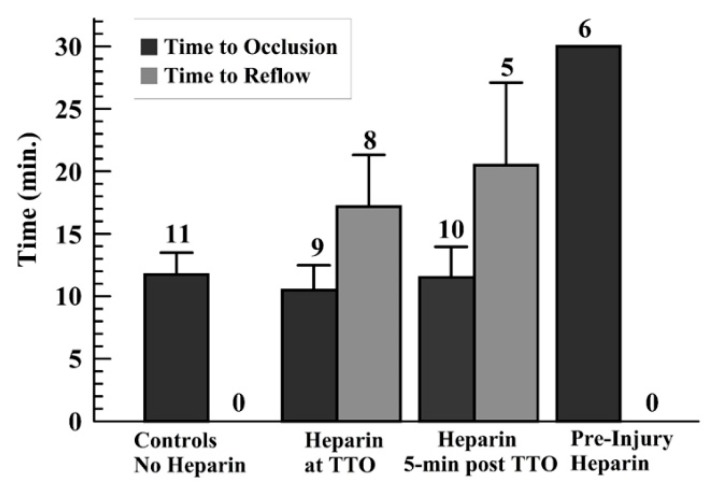
Graph displaying the average and standard deviations for the time to occlusion (TTO) (a), and for the time to reflow (TTR) (b) for those runs wherein reflow occurred above the 10% of baseline flow after TTO, for later-heparin administration groups (middle two sets of groups).

As no occlusion occurred with preinjury heparin (TTO = 30 min—end of monitoring period—for all runs), only the other three groups were analyzed for TTO and TTR. The TTO showed no statistical differences among these other groups (Figure 3). The controls did not show reflow events; thus, a comparison of the TTR for the later-infused heparin groups was done, showing no significant difference in these times (Figure 3). Another way to present the TTO data is to use a “survival curve”, where nonsurvival is defined as occlusion, marked as the TTO; these data are shown for the groups in Supplemental Figure S5.

## 4. Discussion

These findings reveal that, in a ferric chloride model of thrombus induction in rat carotid arteries, the timing of heparin infusion is critical for preventing thrombotic occlusion and promoting return of flow after occlusion. The dose of heparin was chosen to be commensurate to that of a high-dose clinical bolus and based on previous experience with the use of heparin anticoagulation in rats [16,17,18]. When the heparin is delayed after initial reflow and particularly after onset of occlusive phenomena, the effectiveness of thrombotic inhibition is reduced. This may have important ramifications for considering when to administer antithrombotic agents intraoperatively or periprocedurally, where there are clear gaps in our understanding of the onset of clinical thrombotic occlusion and how best to practice interventional therapy. The ability to identify conditions of imminent thrombotic occlusion may be critical for the application of such therapy.

The rat ferric chloride model has been used in a number of studies to evaluate thrombosis, particularly in the context of antithrombotic therapies. The controls (no therapy) in our study had an average TTO of ~12 min; other studies using various parameters of ferric chloride concentration and time of application, as well as filter paper dimensions, have control TTO data from 7.8–8.7 min [19,20] to greater than 30 min [8,21,22], with many values between these times [23,24,25,26,27,28,29,30,31,32,33]. We selected our particular ferric chloride induction parameters to provide a moderate injury so that heparin therapy might have an influence, as was determined to be the case. Use of this rat ferric chloride model provides a simple and relatively consistent way to evaluate small artery thrombosis, as it may pertain to clinical thrombosis in the coronary artery.

Despite the wide use of thrombin inhibitors for short-term antithrombotic therapy in coronary artery procedures, little is known about the specific timing of administration with respect to the initiation of and subsequent progression to occlusion of thrombogenesis. Our findings suggest that this timing may be critical and that late administration may be less effective than if given at the earliest signs of potential or impending occlusive thrombus formation. We selected heparin for use in this study because of its history as a reversible antithrombotic agent (via protamine administration). Low-molecular-weight heparins and other agents may have similar effects, requiring earlier administration to prevent thrombotic occlusion.

Limitations of this study include the contrived model of injury, a chemical induction of ferric chloride to the surface of an artery [34]. This contrasts with the mechanical injury associated with both balloon- and stent-associated injuries and with the microvascular anastomoses of coronary artery bypass grafting, wherein the balloon, stent, suture injury and vessel edge exposure present collagen and tissue factor to the reflowing blood, creating the thrombogenic stimulus [10,35]. Despite this difference in thrombotic mechanism, once thrombogenesis begins, the propagation of thrombosis is mediated by the accruing clotting elements—activated platelets and coagulation complex assemblies—which lead to the rapid onset of vessel occlusion through platelet aggregation supported by fibrin matrix assembly. These clotting elements have been shown to develop under both free-radical-based injuries and microvascular anastomoses, with similar time-related onset and progression, shown by real-time intravital fluorescence imaging [12,36]. Thus, a developing thrombus appears to propagate similarly under a variety of thrombus initiation conditions, supporting the relevance to clinical thrombosis of thrombotic occlusion associated with this experimental ferric chloride mechanism of injury.

These findings may provide insight for guiding the use of heparin and other antithrombotic therapies in the context of coronary artery procedures. The current study suggests caution in using heparin therapy after occlusion has been identified, supporting early treatment as more effective. Whether the results of this study are of relevance to clinical prevention of thrombosis through heparin or similar therapy will need further investigation.

## Supplementary Materials

The following are available online at https://www.mdpi.com/2308-3425/7/1/10/s1, Figure S1: Supplemental Figure S1. All traces for the preinjury heparin series (Group 1), using a normalized baseline value of 1 for all traces, regardless of initial baseline flow (which varied among animals). None of the vessels showed signs of occlusion. Figure S2: All data traces for the group with heparin infused at the TTO (Group 2), using a normalized baseline value of 1 for all traces, regardless of initial baseline flow (which varied among animals). Figure S3: All data traces for the group with heparin infused 5 min after the TTO (Group 3), using the same criteria for graphing as in Supplemental Figure S1. Figure S4: All data traces for the control group (saline infused) at the time of TTO (Group 4), using the same criteria for graphing as in Supplemental Figure S1. Figure S5: “Survival Curve” style of graphic portrayal of the TTO data, stepping down the group number (%) as occlusion occurred (time) in each group. The TTR was not included as this created a more chaotic portrayal due to multiple reflow events in some lines after the first reflow event (TTR), causing a lot of up/down direction changes.
