# Supplementary material for: Timing of Heparin Administration Modulates Arterial Occlusive Thrombotic Response in Rats
Source: J Cardiovasc Dev Dis. 2020 Mar 18;7(1):10. doi: 10.3390/jcdd7010010 (PMC7151218; doi:10.3390/jcdd7010010)
Supplement: Supplementary file 1 [file jcdd-07-00010-s001.pdf]

Supplemental Materials

Timing of Heparin Administration Modulates Arterial Occlusive Thrombotic Response in Rats

Amanda B. Matrai [1,] Bryn Kastetter [1] and Brian C. Cooley[1,2,*]

[1] McAllister Heart Institute, University of North Carolina at Chapel Hill, Chapel Hill, NC 27599, USA;

[2] Department of Pathology and Laboratory Medicine, University of North Carolina, Chapel Hill, NC 27599,

* Correspondence: bccooley@email.unc.edu

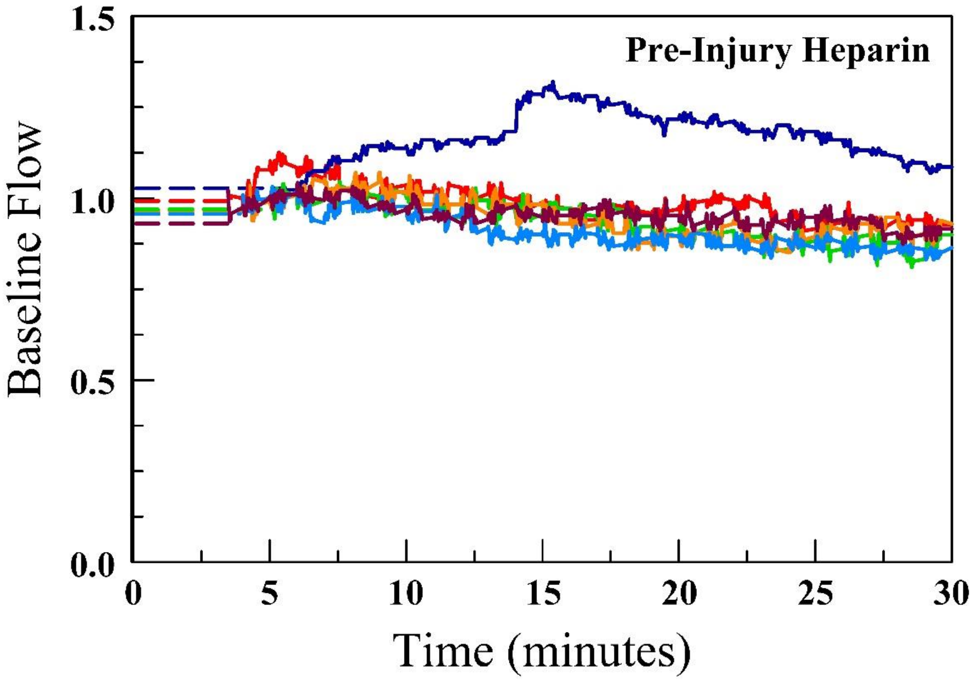

Supplemental Figure S1. All traces for the pre-injury heparin series (Group 1), using a normalized baseline value of "1" for all traces, regardless of initial baseline flow (which varied among animals). None of the vessels showed signs of occlusion.

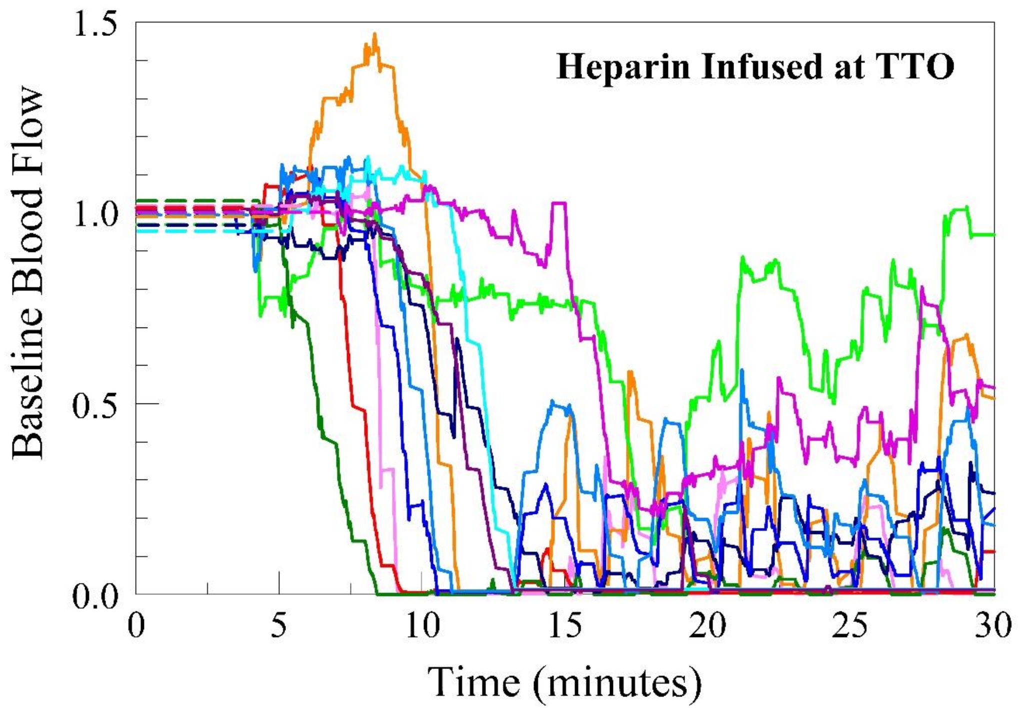

Supplemental Figure S2. All data traces for the group with heparin infused at the TTO (Group 2), using a normalized baseline value of "1" for all traces, regardless of initial baseline flow (which varied among animals).

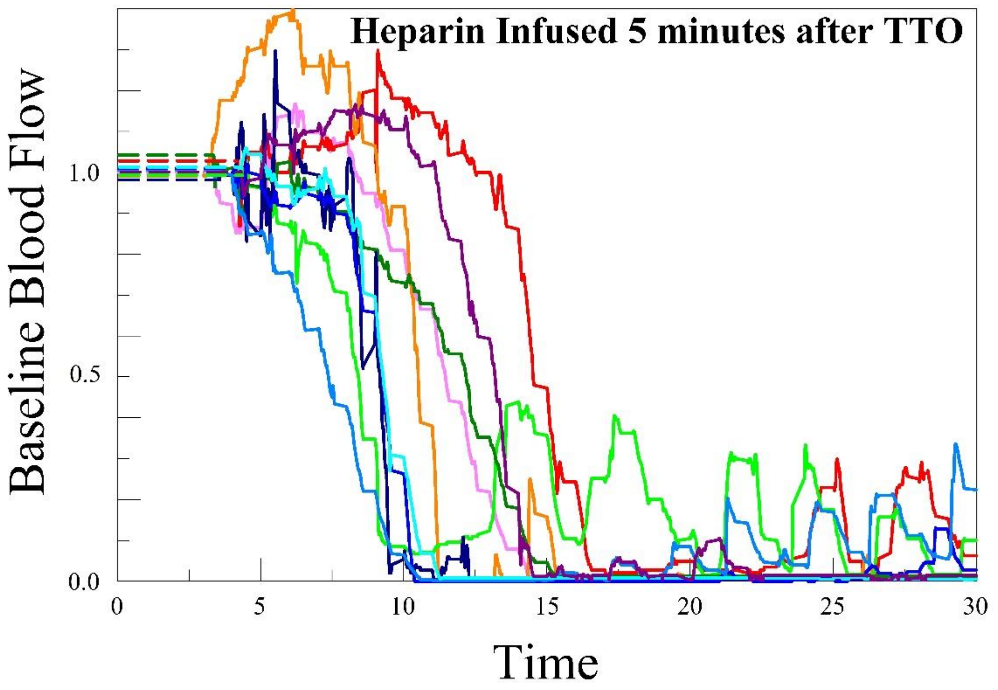

Supplemental Figure S3. All data traces for the group with heparin infused 5 minutes after the TTO (Group 3), using the same criteria for graphing as in Supplemental Figure 1. .

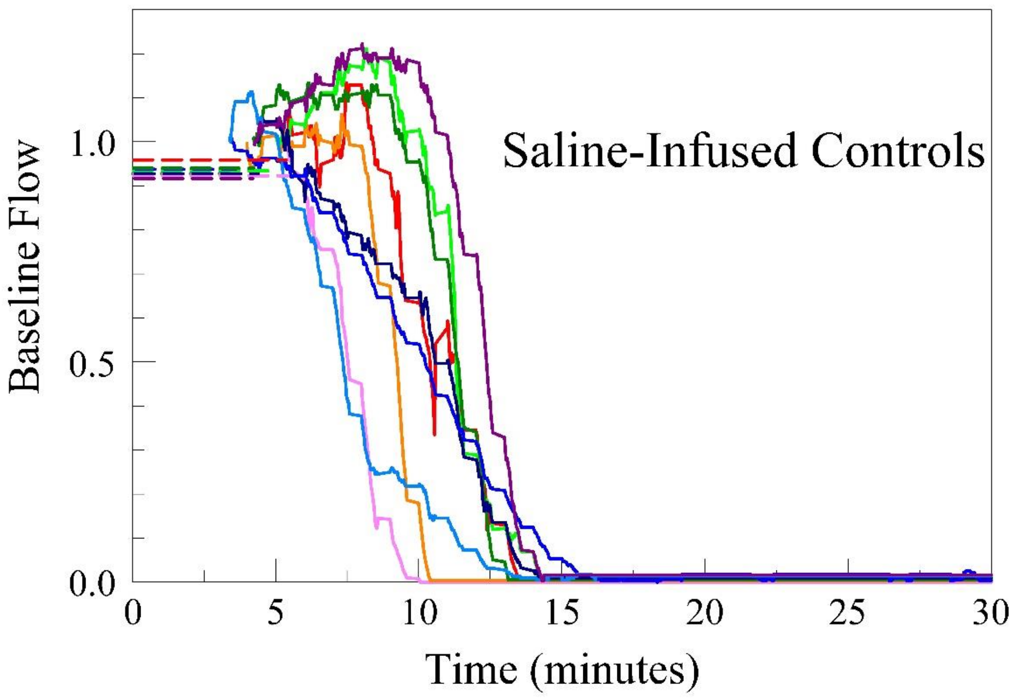

Supplemental Figure S4. All data traces for the control group (saline infused at the time of TTO (Group 4), using the same criteria for graphing as in Supplemental Figure 1. .

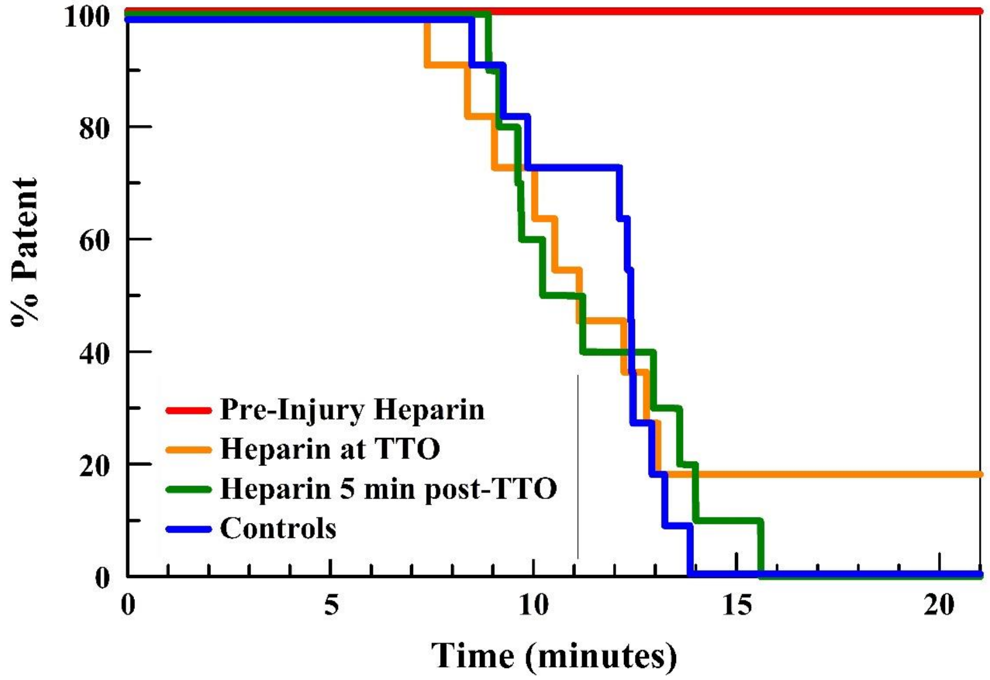

Supplemental Figure S5. "Survival Curve" style of graphic portrayal of the TTO data, stepping down the group number (%) as occlusion occurred (time) in each group. The TTR was not included as this created a more chaotic portrayal due to multiple reflow events in some lines after the first reflow event (TTR), causing a lot of up/down direction changes.
